# Dosimetric, mechanical, and geometric verification of conformal dynamic arc treatment

**DOI:** 10.1120/jacmp.v4i3.2515

**Published:** 2003-06-01

**Authors:** T. Malatesta, V. Landoni, S. delle Canne, A. Bufacchi, L. Marmiroli, O. Caspiani, A. Bonanni, F. Tortoreto, M. V. Leone, R. Capparella, R. Fragomeni, L. Begnozzi

**Affiliations:** ^1^ AFaR, Medical Physics Department S. Giovanni Calibita, Fatebenefratelli Hospital Isola Tiberina n. 39, 00186 Roma Italy; ^2^ AFaR, Radiation Oncology Department S. Giovanni Calibita, Fatebenefratelli Hospital Isola Tiberina n. 39, 00186 Roma Italy

**Keywords:** conformal dynamic arc, dosimetric verification, geometric verification

## Abstract

A conformal dynamic arc (CD‐arc) technique has been implemented at the S. Giovanni Calibita‐Fatebenefratelli Hospital Radiotherapy Center. This technique is performed by rotational beams and a dynamic multileaf collimator (DMLC): during the treatment delivery the gantry rotates and the field shape, formed by the DMLC changes continuously. The aim of this study was to perform dosimetric, mechanical, and geometric verification to ensure that the dose calculated by a commercial treatment planning system and administered to the patient was correct, before and during the clinical use of this technique. Absolute dose values, at the isocenter and at other points placed in dose heterogeneity zone, have been verified with an ionization chamber in a solid homogeneous phantom. In uniform dose regions measured dose values resulted in agreements with the calculated doses within 2%. Isodose distributions have also been determined by radiographic films and compared with those predicted by the planning system. Distance to agreement between calculated and measured isodoses in dose gradient zone was within 2 mm. In conclusion, our results demonstrated the feasibility and the accuracy of the CD‐arc technique for achieving highly conformal dose distributions. Up till now 20 patients have been treated with CD‐arc therapy.

PACS number(s): 87.53.Kn, 87.53.Dq, 87.53.Oq, 87.53.St, 87.53.Tf

## INTRODUCTION

The types of dynamic treatments currently available at our center are: arc, enhanced dynamic wedge (EDW), and treatments using a dynamic multileaf collimator (DMLC), which are intensity modulated (IMRT) and conformal dynamic arc (CD‐arc) treatments. They are all based on a concept of segmentation treatment tables (STT) and different formats of STT are used for the different treatment modalities mentioned above. The fundamental concept is that in dynamic treatments the only information that goes into the delivery system is the MUs versus position; that is, in the case of CD‐arc: the number of monitor units (MUs) versus the MLC shape, leaf position, jaw position, and gantry position. Dose distribution does not depend on dose rate or motion speed as long as the dose versus position relationship is respected. Therefore, in the case of CD‐arc treatments the STT are generated by the CT based treatment planning system and sent to the VARiS database, where they are then available at the treatment unit console.

The principle of the classical arc therapy is the use of multiple fields to reduce the dose to normal tissue and at the same time to integrate to a higher dose throughout the tumor volume itself. The rotational therapy was mainly developed for stereotactic radiosurgery of small cranial lesions. The conventional approach makes use of non‐co‐planar circular arcs with multiple isocenters and circular collimators in order to treat irregularly shaped lesions.^1^ The introduction of computer controlled treatment machines with the dynamic MLC, which allow dynamic adjustment of the field shape with the rotation of the beam, has made it possible to improve the stereotictic radiosurgery technique^2,^, ^3^ and to re‐evaluate rotational therapy for standard radiotherapy called CD‐arc therapy.^4^ The further development of the CD‐arc therapy is the intensity modulated arc therapy (IMAT), where an inverse planning algorithm is used to optimize the number of arc segments and their weights.^5–^, ^9^

In this study problems related to the clinical application of the CD‐arc technique have been investigated, in particular the aim was to verify the dose distribution predicted by the treatment planning system (TPS) and to prepare a suitable program of mechanical and geometric verifications before starting the use of the CD‐arc technique in clinical practice. For some CD‐arc treatments, absorbed dose values measured by the ionization chamber at the isocenter and at fixed points in homogeneous solid phantoms have been compared with the ones calculated, in the same points, by the TPS. For the same treatments, calculated dose distribution has been compared with the experimental one determined, in the above‐mentioned homogeneous solid phantoms, by calibrated radiographic films.

## METHODS AND MATERIALS

CD‐arc therapy has been implemented at the S. Giovanni Calibita‐Fatebenefratelli Hospital Radiotherapy Center. The technique is performed by two high energy Varian linear accelerators 2100 C/D, supplied with the Varian Mark II 80 dynamic MLC (DMLC), while the treatment plan is computed by the TPS CadPlan version 6.3.5.

To deliver CD‐arc therapy particular attention has to be paid to the properties of DMLC^7^ and its correct configuration in the TPS CadPlan. The DMLC is mounted below the conventional block collimators and it consists of 40 pairs of tungsten alloy leaves of 1 cm width at isocenter. The range of travel of each individual leaf is 15 cm at isocenter relative to the carriage; for DMLC operation the carriages remain fixed during dose delivery. Since CadPlan TPS requires a single leaf transmission value for each energy, it has been obtained as the average of midleaf transmission and interleaf leakage: the values achieved were 1.2% and 1.7% for 6 MV and 15 MV respectively. The position of the DMLC during the dynamic arc therapy is established by the TPS that automatically designs, for a given margin, the beam shape around the PTV for each gantry angle but, if it is necessary, the leaf positions can also be corrected manually. The dynamic MLC parameters are defined in the treatment unit configuration, and the CadPlan calculation process takes into account the leaf transmission value, rounded leaf end shape and tongue and groove effect. The MLC motion file is generated by the Leaf Motion Calculator (LMC) module, included in the CadPlan software.

Forward planning has been used for the CD‐arc treatment technique. In the calculation arcs are approximated as multiple shaped fields (segments) spaced about every 10° (max^18^ around the patient. The number of segments (up to 20) and the ranges of the arcs are chosen manually. The dose matrix calculation grid of 0.5 cm. The final treatment plan is input into the leaf‐sequencing program to generate the dynamic MLC prescriptions. The treatment delivery is accomplished by programming the linear accelerator to deliver an arc and the DMLC to move through a sequence of fields.

In the dynamic arc therapy the MLC leaves move as a function of the gantry position in the arc. The leaf positions between sequential fields in the DMLC file are linearly interpolated to 0.1° gantry angle for dynamic arc plans to generate intermediate leaf positions in the MLC controller. Each DMLC field file contains the sequence of shape that the MLC is programmed to deliver for the field. When creating a DMLC field file, you do not have to include information about how the MLC moves, its speed, and so on: the MLC application determines these aspects of the treatment. One important feature of the DMLC field file format is that the creator of DMLC field files need not be concerned with MLC kinematics or other details of treatment delivery such as optimal dose rate and leaf speeds. The control system automatically computes an optimal dose rate and MLC speed for each segment so that the field is delivered in the shortest possible time. A basic check on leaf movement rates is performed to see if an arc treatment plan is achievable. With a leaf position tolerance *t*, for any arc segment of *n* degree, no leaf position change *d* can be made, such that: d>(n/0.5)*t.

### A. CD‐ARC mechanical and geometric verification

Recent works have studied the effects of gravitational acceleration and leaf velocity on leaf position accuracy that can significantly affect the dose accuracy during CD‐ARC and intensity modulated arc therapy (IMAT)^7^ delivery. The gravitational effect was tested measuring, with a cylindrical ion chamber, the dose of a uniform field delivered dynamically with a small sweeping gap.^10^ The test was repeated for different gantry angles (0°, 90°, 180°, 240° and 270°) to assess the effect of gravity on the performance of the MLC in dynamic mode; the difference was about 1%, indicating that the gravitational effect can be negligible. The leaf position accuracy decreases at a higher dose rate; at a dose rate of 300 MU/min, that we have chosen to use, the average leaf position uncertanity is 0.14 cm.^11^.

During MLC initialization, a primary optical calibration and a secondary mechanical verification system provides an absolute calibration for the spatial position of each MLC leaf to within ±0.1 mm. The MLC leaf position tolerance, measured at isocenter during gantry rotation, can be set between 0.5 mm and 5.0 mm. In the sliding window technique of the DMLC application, the delivered dose is directly related to the gap between opposed leaves. The dose error is large for small gap width and large gap error.^10^ In general, while for clinical IMRT treatments the gaps are less than 1 cm, for CD‐arc treatments the gaps are greater than 3 cm; therefore, a strict leaf position tolerance is not mandatory. Moreover, considering that the increase of the tolerance value increases the treatment time,^10^ 1 mm tolerance has been considered an adequate value to be set. If this limit is exceeded during treatment delivery the linac controller generates an interlock that stops the beam.

At the end of the CD‐arc treatment delivery the linac console shows the dose difference standard deviation (SD) between the actual dose and the desired dose which shall be <0.20 MU and the dose weighted gantry position difference standard deviation (SPD) between the actual gantry position and the desired gantry position which shall be <0.5.

For each patient, before starting the real treatment, in absence of the patient but with the personalized immobilizer fixed to the couch, a test run has been conducted to assess the geometric and the dosimetric accuracy. Indeed during the test CD‐ARC delivery, every 50 ms, the difference between the actual and the planned position of each MLC leaf is stored in a MLC log file so that the treatment mechanical accuracy can be checked. Moreover, every 50 ms, during the test treatment delivery, as well as during real treatment, the linac controls for each angle the MUs delivered versus the gantry position and at the end it supplies the calculated standard deviation.

To verify that the agreement between leaf positions during treatment and that generated by CadPlan TPS, within the established tolerance of 1 mm, light fields of different arc segments of the CD‐arc therapy have been compared with isocentric CadPlan beam's eye view prints. Therefore a verification of the complete treatment delivery, as it has been planned, can be performed before treatment.

### B. CD‐ARC dosimetric verification

In order to verify the accuracy of the TPS calculated dose before starting to deliver CD‐arc technique, the following dosimetric verifications have been planned and performed in a solid RW3 plastic phantom:

(i) Absolute dose values in different points have been measured by a Farmer NE type 2581 ionization chamber connected to a NE 2670 electrometer and compared with the planned dose values obtained as the average dose calculated within the active volume of the chamber.

(ii) Planar dose distributions have been verified, by Kodak EDR‐2 radiographic films. Films have been irradiated during CD‐arc treatment, in the homogeneous plastic phantom reproducing patient main dimensions. The optical densities have been determined by the Vidar VXR‐12 film digitizer and converted in dose values by calibration dose curves. New calibration curves were established for each measurement batch; the EDR‐2 films were irradiated with dose values up to about 6 Gy. The RIT113 Image Processing Software was used to compare the experimental normalized dose distribution with the CadPlan calculated ones.

### C. Dosimetric verification No. 1

Absolute dose values have been determined in an homogeneous RW3 plastic phantom, 30 cm ×30 cm×20 cm in dimensions, by a cylindrical ionization chamber positioned at the isocenter (low gradient zone) in order to verify CadPlan calculated dose values in this point.

**Figure 1:**
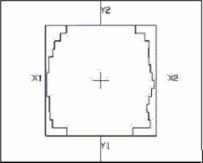
(Color) 2D beam's eye view of a field with jaw settings of 14 cm×14 cm and the MLC shielding about the 10% of the field area.

The isocenter has been placed at 10 cm depth. A 15 MV photon beam, with jaw settings of 14 cm×14 cm, has been set at the isocenter depth. The verification has been performed for two different arcs respectively of 180° and 360°. For each arc, three different beam conformations have been analyzed: without MLC, with a static MLC and with a dynamic MLC; where applied, the MLC shielding was less than 10% of the beam area (Fig. 1). For each beam configuration a dose of 3 Gy has been prescribed at the isocenter. Results are reported in the Results and Discussion section (Table I).

**Table I:** Comparison between measured and calculated dose (3 Gy) at isocenter delivered by two 15 MV photon arc treatments in three different shielding conditions: without MLC, with a static MLC, and with a DMLC shielding about the 10% of the field area respectively.

	No MLC		Static MLC		Dynamic MLC	
Arc	Dose (Gy)	Deviation (%)	Dose (Gy)	Deviation (%)	Dose (Gy)	Deviation (%)
180°	2.985	−0.5	2.990	−0.3	2.989	−0.3
360°	2.985	−1.4	2.957	−1.4	2.958	−1.4

### D. Dosimetric verification No. 2

**Figure 2:**
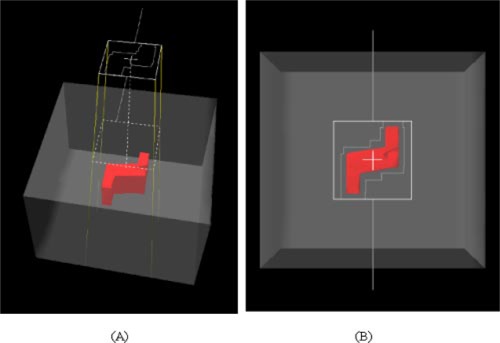
(Color) The 30 cm ×30 cm×20 cm phantom with an S‐shaped PTV: (a) homogeneous phantom, (b) 2D beam's eye view. The MLC shields the 75% of the field area.

An *S*‐shaped PTV illustrated in Fig. 2, in the same phantom 30 cm ×30 cm×20 cm in dimensions, has been used to compare calculated and measured dose at the isocenter. A nominal 15 MV energy photon beam has been used for this test. The isocenter has been placed at 10 cm depth. A field with jaw settings of 12 cm×12 cm has been set at the isocenter. A dose of 1 Gy has been prescribed at the isocenter of the homogeneous phantom.

In order to evaluate by degrees possible factors affecting the dose accuracy, a first comparison has concerned a static beam conformed by a static MLC shielding about the 75% of the beam area [Fig. 2(b)]. Results are reported in the Results and Discussion section Table I(I).

**Table II:** Accuracy verification of dose delivered at isocenter in a static treatment and CD‐arc in homogeneous S phantom. Calculated dose: 1 Gy for static field and 2 Gy for CD‐arc.

Static field		Conformal dynamic arc	
Dose (Gy)	Deviation (%)	Dose (Gy)	Deviation (%)
0.986	−1.4	1.961	−2.0

### E. Dosimetric verification No. 3

Calculated and measured dose at isocenter have been successively compared for a 360° CD‐arc treatment obtained by CadPlan TPS in order to irradiate the above described *S*‐shaped volume. The position of the MLC leaves has been automatically determined by CadPlan by selecting a 0.8 cm margin from the PTV The dose prescription was 2 Gy at the isocenter of the homogeneous phantom as in point 2. For the same configurations, planar dose distribution measurements have been carried out by irradiating an EDR2 Kodak film positioned perpendicularly to the 0–180 axis in the isocenter plane. Results are reported in the Results and Discussion section (Table II, Fig. 4).

**Figure 3:**
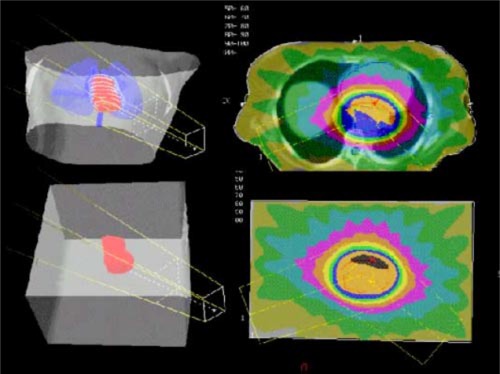
(Color) A mediastinum treated with a CD‐arc of 240° from 250° to 130°, a 15 MV photon beam with a 10 cm×10 cm size and the isocenter set at a 10.5 cm depth. In the upper part of the figure the clinical case is shown, and in the lower part the geometric phantom used for the experimental measurements is shown.

**Figure 4:**
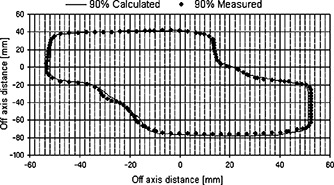
CD‐arc treatment: comparison between measured and calculated 90% isodoses, determined at the isocentric transversal plane inside the homogeneous phantom with an *S*‐shaped PTV. Dose distributions are normalized to the isocentric plane maximum dose value.

### F. Dosimetric verification No. 4

The following measurements have been carried out to verify two clinical CD‐arc treatments before the radiotherapy course start. Measured and calculated point dose and planar relative dose distribution have been compared. Since the CadPlan TPS does not allow the export of the MLC file of a CD‐arc treatment, necessarily the same DMLC was generated again on the homogeneous phantom. The PTV derived from the clinical case has been copied by means of CadPlan digitizer in a homogeneous solid phantom of dimensions as much similar as possible to the three main patient dimensions. The patient treatment plan has been applied to the homogeneous phantom and the volume dose distribution has been calculated so that experimental dose verification could be performed.

The first analyzed clinical case, shown in Fig. 3, has been a mediastinum treated with a 15 MV photon CD‐arc of 240° (from 250° to 130°), with jaw settings of 10 cm×10 cm at the isocenter depth equal to 10.5 cm. The patient thickness was 19.5 cm. Point dose measurements have been performed at the isocenter, where a dose gradient of 1% mm−1 existed, 2 cm above the isocenter, where the dose distribution was homogeneous. A dose of 2 Gy has been prescribed at the isocenter.

The second analyzed clinical case has been a lung tumor treated with a CD arc technique composed of three 15 MV photon beam arcs, differently weighted, respectively of 80° (from 320° to 40°, weight 1.2), of 25° (from 150° to 175°, weight 0.5), and of 8° (from 187° to 205°, weight 0.5), with a 11 cm×7 cm field size at the isocenter depth of 7 cm. Point dose measurements have been performed at the isocenter depth where a dose of 2 Gy has been prescribed. The patient thickness was 24 cm.

## RESULTS AND DISCUSSION

The results of dosimetric verification, the controlled linac accuracy in delivering CD‐arc, and the geometrical accuracy in patient positioning clearly indicates the reliability of the technique so to allow its clinical application. Therefore, the CD‐arc technique has been consequently applied to small volume PTVs located in the thorax or in the pelvis. These PTV's for which CD‐arc technique has been applied usually required a multiple beam conformal technique with five or six beams which needed more time to be applied to the patient. On the other hand, the average time a patient usually spends on the treatment table for a CD‐arc treatment is less than 8 min. Therefore, the first results in the application of the technique indicate the reliability and the reproducibility of the dose delivery and also the possibility to save time without losing accuracy at a very busy treatment unit.

### A. CD‐ARC mechanical and geometric verification

In agreement with leaf position accuracy determined by comparing CadPlan BEV and light field shapes for different conformal segments of DMLC treatments, the interlock due to the leaf position out of the set tolerance (1 mm) has never appeared either before the treatment or during treatment. Moreover, looking at the MLC log file stored during the test run before the treatment the root mean square of the differences between planned and real leaf position was always ≥0.1 mm. In all the treatments performed til now interlock connected to the SD and the SDP has never appeared, indicating that the standard deviation of the dose and position during treatment delivery has never exceeded the tolerance limits of 0.20 MU and 0.50°.

### B. CD‐ARC dosimetric verification

The results related to measurement No. 1 are reported in Table I, whereas the results related to measurements No. 2 and No. 3 are reported in Table II. It is clear that there is no difference between the three arc treatments: without MLC, with static MLC, and with dynamic MLC when considering the comparison between the measured and calculated dose at the isocenter, as shown in Table I. Therefore, no additional uncertainty in dose calculation is added with the use of DMLC in arc therapy. The deviation increases with increasing the arc length but remains within 2%. Results shown in Table II indicate that with CD‐arc treatment the measured dose in points is within 2% in comparison with the planned one and no relevant increase in deviation is introduced with the use of CD‐arc in comparison with the static field. All the measured dose values were slightly lower than the corresponding planned dose values because of a systematic error in dose calculation of about 1%.

Figure 4 shows the CD‐arc treatment comparison between measured and calculated isodoses in the isocenter plane, inside the homogeneous phantom with the *S*‐shaped PTV Distance to agreement between calculated and measured 90% isodoses is less than 2 mm. The measured isodose appears to follow the discrete profile of the MLC leaves end especially where the target profile is oblique with respect to the MLC leaves end. In the calculated isodose this effect is less evident because the grid calculation dimension of 0.5 cm is comparable with the MLC leaf dimension (1 cm at isocenter).

**Figure 5:**
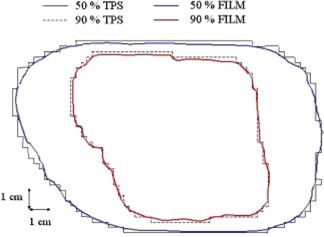
(Color) CD‐arc treatment of a mediastinum: comparison of measured and calculated 90% and 50% isodoses with dose distribution normalized to the maximum, at the isocenter plane.

Figure 5 shows the CD‐arc treatment comparison between measured and calculated 90% and 50% isodoses, with dose distributions normalized to the maximum, relative to the first clinical case. Distance to agreement between calculated and measured isodose lines were less than 2 mm. Point dose values of 1.970 Gy(−1.5%) at isocenter and 1.991 Gy(−0.5%) in the point inside the uniform dose region 2 cm above the isocenter were measured, versus calculated doses of 2 Gy: the dose differences in uniform regions is within 2%.

**Figure 6:**
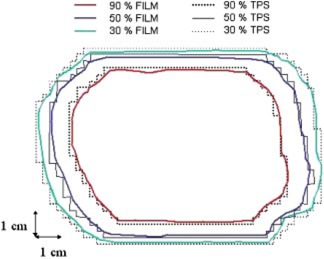
(Color) CD‐arc treatment of a lung tumor: comparison of measured and calculated isodoses, with dose distribution normalized to the maximum, at the isocenter plane: 90%, 50%, and 30% isodoses are reported.

Figure 6 shows the comparison between measured and calculated isodose distributions at 90%, 50%, and 30% relative to the second clinical case: the distance to agreement is less than 2 mm. The associated measured point dose at isocenter is 3.014 Gy, that is +0.5% greater than the TPS calculated value of 3 Gy.

## CONCLUSIONS

For different experimental conditions an excellent agreement between the isodoses measured by radiographic films and those calculated by the TPS has been demonstrated, indeed the distance to agreement between the 90%, 50%, and 30% calculated and measured isodoses was within 2 mm. Moreover, in uniform dose regions, the point dose measurements in homogeneous phantom agreed with the calculated doses within 2%. The tests that have been applied to verify the CD‐arc treatment accuracy checked both the mechanical DMLC leaves position and the treatment delivery accuracy.

In conclusion, results demonstrated the feasibility and the accuracy of the CD‐arc that is a relatively new and time‐sparing dynamic technique and that is capable of providing highly conformal dose distributions. Moreover, the introduction in the clinical practice of this technique did not increase the number of maintenance inspections to the linac. The dynamic treatments have never been stopped and no additional problems arose due to their use.

## References

[1] D. D. Leavitt , F. A. Gibbs, Jr. , M. P. Heilbrun , J. H. Moeller , and G. A. Takach, Jr. , “Dynamic field shaping to optimize stereotactic radiosurgery,” Int. J. Radiat. Oncol., Biol., Phys. 21, 1247–1255 (1991). 1938523 10.1016/0360-3016(91)90283-a

[2] D. D. Leavitt , M. Tobler , D. Gaffney , P. Zhang , and J. Moeller , “Comparison of interpolated vs. calculated micromultileaf settings in dynamic conformal arc treatment,” Med. Dosim 25, 17–21 (2000). 10751714 10.1016/s0958-3947(99)00035-7

[3] T. D. Solberg , K. L. Boedeker , R. Fogg , M. T. Selch , and A. A. DeSalles , “Dynamic arc radiosurgery field shaping: a comparison with static field conformal and noncoplanar circular arcs,” Int. J. Radiat. Oncol., Biol., Phys. 49, 1481–1491 (2001). 11286857 10.1016/s0360-3016(00)01537-6

[4] M. Tobler , G. Watson , and D. D. Leavitt , “The application of dynamic field shaping and dynamic dose rate control in conformal rotational treatment of the prostate,” Med. Dosim 27, 251–254 (2002). 12521068 10.1016/s0958-3947(02)00148-6

[5] C. X. Yu , X. A. Li , L. Ma , D. Shepard , M. Sarfaraz , T. Holmes , M. Suntharalingam , and C. Mansfield , “Clinical implementation if intensity‐modulated arc therapy,” Int. J. Radiat. Oncol., Biol., Phys. 48, 215–219 (2000). 10.1016/s0360-3016(02)02777-3 12023150

[6] X. A. Li , L. Ma , C. X. Yu , S. Naqvi , and T. Holmes , “Monte Carlo dose verification for intensity modulated arc therapy,” Int. J. Radiat. Oncol., Biol., Phys. 48, 216–219 (2000).

[7] L. Ma , C. Yu , M. Sarfaraz , T. Holmes , D. Shepard , A. Li , S. DiBiase , P. Amin , M. Suntharalingam , and C. Mansfield , “Simplified intensity modulated arc therapy for prostate cancer treatments,” Int. J. Radiat. Oncol., Biol., Phys. 48, 350–351 (2000).

[8] E. Pignoli , S. Serretiello , A. Somigliana , G. Zonca , R. Pellegrini , V. Mongioj , and R. Marchesini , “Dosimetric verification of a commercial 3D treatment planning system for conformal radiotherapy with dynamic multileaf collimator,” Phys. Med. Biol. 45, 77–84 (2000). 10.1088/0031-9155/45/8/401 10958205

[9] M. Tobler , G. Watson , and D. D. Leavitt , “Intensity‐modulated photon arc therapy for treatment of pleural mesothelioma,” Med. Dosim 27, 255–259 (2002). 12521069 10.1016/s0958-3947(02)00149-8

[10] T. LoSasso , C. S. Chui , and C. C. Ling , “Physical and dosimetric aspects of a multileaf collimation system used in the dynamic mode for implementing intensity modulated radiotherapy,” Med. Phys. 25, 1919–1827 (1998). 9800699 10.1118/1.598381

[11] C. R. Ramsey *et al.*, “Leaf position error during CD‐arc and intensity modulated arc treatment,” Med. Phys. 28, 67–72 (2001). 11213924 10.1118/1.1333410

